# The Use of Natural Health Products Especially Papaya Leaf Extract and Dandelion Root Extract in Previously Untreated Chronic Myelomonocytic Leukemia

**DOI:** 10.1155/2018/7267920

**Published:** 2018-12-18

**Authors:** Leena T. Rahmat, Lloyd E. Damon

**Affiliations:** ^1^Department of Hematologic Malignancies, Johns Hopkins University, The Sidney Kimmel Cancer Institute, Sibley Memorial Hospital, Washington, DC, USA; ^2^Department of Hematologic Malignancies and Blood and Marrow Transplant, University of California, San Francisco, CA, USA

## Abstract

Chronic myelomonocytic leukemia (CMML) is a clonal hematopoietic disorder which shares clinical and morphological features of myelodysplastic syndrome and myeloproliferative neoplasms. Conventional therapeutic options include hydroxyurea, hypomethylating agents, and systemic chemotherapy, which are all palliative measures and are associated with potential side effects. Allogeneic hematopoietic cell transplantation is the only curative option. Natural health products such as papaya leaf extract and dandelion root extract have been shown to demonstrate anticancer activity in preclinical and clinical studies, respectively. We present a case study of a 76-year-old male with previously untreated CMML, whose hematological parameters remained stable and whose bone marrow blast counts vastly improved while taking papaya leaf extract and dandelion root extract.

## 1. Introduction

Chronic myelomonocytic leukemia (CMML) is an aggressive and generally resistant form of hematopoietic stem cell neoplasm with the potential of progression to an acute myelogenous leukemia and with a median survival of 12 to 24 months from diagnosis [1]. Because of lack of CMML-specific clinical trials, the optimal treatment of CMML is unclear. Allogeneic hematopoietic cell transplantation (alloHCT) is the only disease-modifying therapy, but many patients are not candidates for an alloHCT due to multiple comorbidities and/or advanced age. Numerous anticancer therapeutic agents have been derived from natural products [2]. Research into the antimalignancy properties of natural health products (NHPs) dates back to the 1980s. A handful of preclinical studies have demonstrated anticancer activity with the use of papaya leaf extra (PLE) and dandelion root extract (DRE) [1, 2].

## 2. Case

A 76-year-old Caucasian male with a history of stage Ic prostate cancer, gleason 3 + 3, status after radiotherapy and antiandrogen therapy in 1998 (in remission), hypertension, and IgG2/IgG4 subclass deficiency presented with CMML-1 with deletion 7q (q22q32) in 11 of 20 cells (55%) diagnosed in December 2008. JAK 2 mutation analysis and quantitative RT-PCR for the BCR-ABL transcript were negative. He had an isolated thrombocytopenia at presentation, his diagnostic bone marrow biopsy demonstrated trilineage hematopoiesis without dysplasia and 1% blasts, and atypical intermediate-sized monocytoid cells increased at 20%–30% of the bone marrow cellularity. No systemic treatment was commenced at the time. His other comorbidities include gastroesophageal reflux disease, lumbar spinal stenosis (L3–L4), and lumbar neuroforaminal narrowing due to degenerative disc disease.

In early 2009, he started PLE in the form of papaya leaf tea, 4 grams once daily in the morning and one teaspoon of elixir at night. He also had IgG2 and IgG4 subclass deficiencies. For his IgG subclass deficiencies, he was treated with cimetidine 400 mg 2 times daily starting from October 2014. He also started DRE 520 mg capsules, two capsules once daily in early 2015. His bone marrow blasts peaked at 11% in October 2009 and have been less than 5% since March 2013. Of note, both the papaya leaf extract and DRE supplements were commercial products.

Prior to his diagnosis of CMML, he had been on numerous over-the-counter supplements (commercial products): pomegranate XT 1 mg daily; vitamin C 500 mg daily; L-carnitine, red yeast rice, niacin, vitamin B6, and omega-3 fatty acids (all started in the 1990s); nettle root daily, pyrroloquinoline 1 tablet daily, melatonin 1.5 mg daily, beta-glucan 400 mg daily, and ellagic acid (all started in 2000); and one red rooibos tea bag daily with the PLE tea. Following the diagnosis of CMML, he started numerous additional OTC supplements: vitamin K daily (2011); bio-curcumin daily and vinpocetine 10 mg daily (2011); inositol 500 mg daily (2012); resveratrol 1 tab bid, vitamin B12, folic acid, and boswellia 100 mg daily (2013); *S*-adenosylmethionine (SAM-E) (2014) and rice bran 500 mg bid (2014); and maitake mushroom elixir about 2–3 cc once daily (2017). A trend of his blood counts and bone marrow results are illustrated in Table 1.

The patient had no side effects and no worsening of comorbidities attributable to the supplements. He was hospitalized in June 2017 with acute hypoxemic respiratory failure and reactive airways treated with antibiotics and bronchodilators, which was not attributed to the supplements. Additionally, he was not neutropenic (ANC was always greater than 1200 cells/*μ*L) during the hospitalization. Clinically, he continues to feel well and his hematological parameters remain consistently stable.

## 3. Discussion

The World Health Organization (WHO) has classified CMML as a myelodysplastic syndrome (MDS) overlapping with a myeloproliferative disease (MPD) [3]. Its dismal prognosis indicates a pressing need for more effective alternatives therapies. The use of NHPs may well provide a nontoxic and less-expensive therapeutic alternative. Our hypothesis is that our patient's CMML disease course has been modified by NHPs and OTC supplements. In vitro data demonstrate anticancer activity with the use of PLE, DRE, and curcumin [1, 4–6]. It is unclear whether any of the other supplements have contributed to the stable disease course.

Nguyen et al. conducted a literature review on DRE (*Carica papaya*) which was noted to have anti-inflammatory and anticancer effects [4]. Otsuki et al. demonstrated a significant growth inhibitory effect of the *Carica papaya* extract on tumor cell lines [5]. They also demonstrated a reduction in cytokines IL-2 and IL-4 in peripheral blood mononuclear cells [5]. There are most interesting findings; the PLE tea and elixir our patient consumed may have had an antiproliferative effect on his CMML.

One study demonstrated a selective efficacy of DRE (*Taraxacum officinale*) in inducing apoptosis in CMML cell lines [1]. DRE is a NHP proposed to contain antioxidant properties; however, the exact mechanism of action in cancer cells remains elusive [2]. Studies of DRE have demonstrated a selective inhibition of the death receptor-mediated pathway of apoptosis [2]. An additional widely used NHP is turmeric (*Curcuma longa*) whose active ingredient is curcumin which has been studied for antimalignancy effects.

Arber et al. demonstrated selective efficacy in induction of programmed cell death in aggressive and resistant CMML cell lines using DRE [3]. Hamm et al. described a case of a 70-year-old man with a history of primary refractory acute myelomonocytic leukemia who received DRE tea after failing induction chemotherapy [6]. What emerged was a diagnosis of CMML. The patient achieved a complete hematological remission with the ongoing use of DRE tea. When he drank less than three cups per day of the DRE tea, his peripheral blood monocyte count started to rise [6]. The same group reported an elderly female with CMML who only used DRE tea to treat her disease. She achieved a hematological remission but relapsed 3 months later [6]. This group reported a third case of a 60-year-old female with primary refractory acute myelomonocytic leukemia and a subsequent diagnosis of CMML who achieved a hematological remission after starting DRE tea, three cups per day. She remains in complete hematological remission at least 5 months after starting DRE tea. A fourth patient with acute myeloid leukemia (AML), who was not a candidate for intense systemic chemotherapy, was treated with low-dose cytarabine followed by DRE tea. She developed peripheral blasts when she stopped the tea for one month, but she continues the DRE tea 15 months from the AML relapse [6].

## 4. Conclusion

NHPs may well provide a potential nontoxic therapeutic alternative to conventional systemic therapy in the treatment of CMML. Our patient remains on numerous NHPs, most notably on PLE tea and DRE. He remains asymptomatic, transfusion independent with stable counts. DRE and PLE may provide an improved quality of life of patients with CMML if systemic chemotherapy can be avoided.

## Figures and Tables

**Table 1:** 

Month/year	Bone marrow blast %	FISH deletion 7q % metaphases or standard banding karyotype (no. of positive cells/number of metaphase examined)	WBC (k/*µ*L)	Hb (g/dL)	Platelet (k/*µ*L)	ANC (k/*µ*L)	AMC (k/*µ*L)	IgA (mg/dL)	IgG (mg/dL)	IgM (mg/dL)
12/2008	1	11/20	4.7	14.7	122	1.91	1.51			
05/2009	8		4.3	15	101	1.55	1.38			

10/2009	11	52 (13/20)	3.4	15.8	75	0.94	1.43			
03/2010	8.5	(9/20)	5	16.5	73	1.5	1.76			

10/2010	4	75	4.5	17.2	88	1.25	1.99			
12/2011			4.9	16.5		1.08	1.71			

03/2012	8	75	4.2	16.7	70	1.34	1.80			

03/2013	<5	70	4	16.1	61	0.82	1.97			

03/2014	<5	89	4.2	15.9	73	0.86	2.27			
03/2015	5	95	5.2	15.4	84	1.56	2.55	111	661	59.3

03/2016	3	93	4.8	15.3	71	1.41	2.03	106	637	49.1
03/2017	4.8	100	5.5	16.2	70	1.68	2.25			

09/2017			7.2	15	70	2.16	3.5	100	572	50.3
12/2017			5.1	14.8	76	1.29	4.67	130	659	51.2
